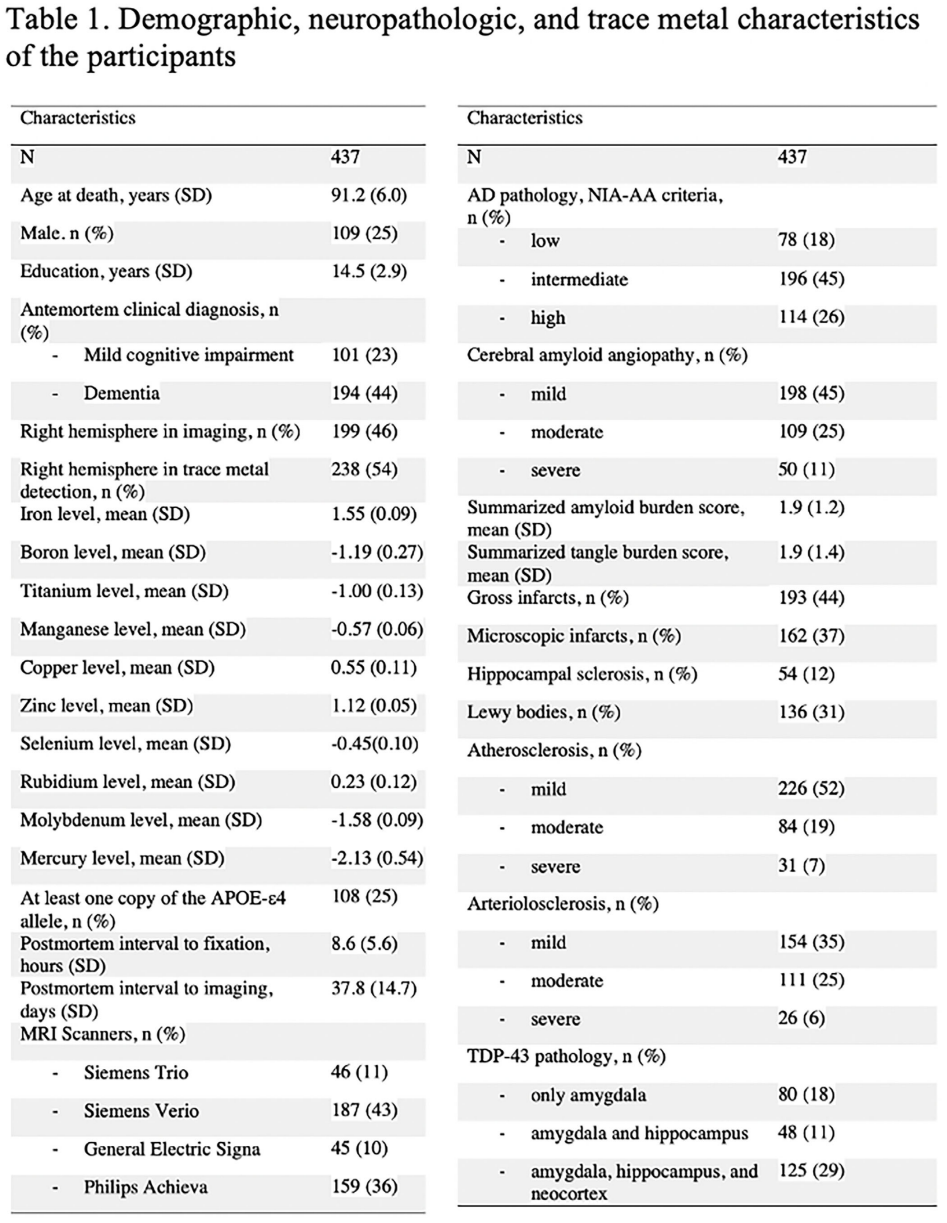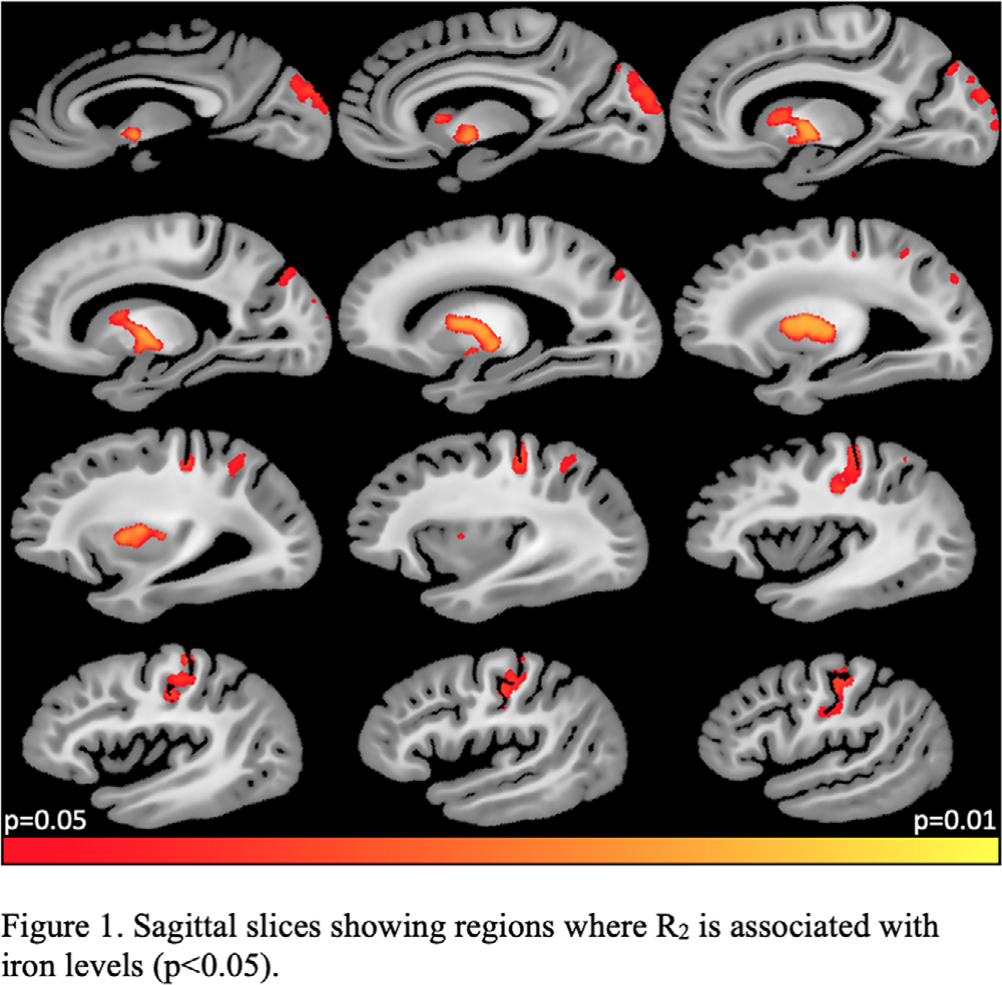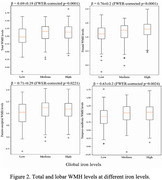# Elevated iron levels in the older adult brain are associated with higher R2 relaxation rate in gray matter and more white matter hyperintensities: An ex‐vivo MRI, pathology and mass spectrometry study

**DOI:** 10.1002/alz.093668

**Published:** 2025-01-09

**Authors:** Md Tahmid Yasar, Ashley I. Bush, Scott Ayton, Puja Agarwal, Sonal Agrawal, David A. Bennett, Julie A. Schneider, Konstantinos Arfanakis

**Affiliations:** ^1^ Illinois Institute of Technology, Chicago, IL USA; ^2^ The Florey Institute of Neuroscience and Mental Health, The University of Melbourne, Australia, Melbourne, VIC Australia; ^3^ Rush Alzheimer's Disease Center, Rush University Medical Center, Chicago, IL USA

## Abstract

**Background:**

Elevated iron in brain is a source of free radicals that causes oxidative stress which has been linked to neuropathologies and cognitive impairment among older adults. The aim of this study was to investigate the association of iron levels with transverse relaxation rate, R2, and white matter hyperintensities (WMH), independent of the effects of other metals and age‐related neuropathologies.

**Method:**

Cerebral hemispheres from 437 older adults participating in the Rush Memory and Aging Project study (Table 1) were imaged ex‐vivo using 3T MRI scanners. R2 maps were generated from multi‐echo spin‐echo data and then registered to an ex‐vivo brain template. WMH were segmented based on T2‐weighted images. WMH volume was normalized by the total hemisphere volume and then log‐transformed. Following ex‐vivo MRI, all hemispheres underwent neuropathologic assessment including Aß plaques, neurofibrillary tangles, LATE‐NC, hippocampal sclerosis, Lewy bodies, cerebral amyloid angiopathy, gross infarcts, microscopic infarcts, atherosclerosis, and arteriolosclerosis (Table 1). Inductively coupled plasma mass spectrometry was used to measure brain trace metal levels. The assessed metals included iron, boron, titanium, manganese, copper, zinc, selenium, rubidium, molybdenum, and mercury (Table 1). Linear regression was used to test the association of R2 and WMH burden with iron levels. All models were controlled for all other metals and neuropathologies listed above, demographics (age at death, sex, years of education), the presence of the APOE‐e4 allele, postmortem interval to fixation and to imaging, and scanner. Statistical analysis was performed using PALM, with tail‐accelerated 5,000 permutations. Statistical significance was set at p<0.05 after family wise error rate correction.

**Result:**

The voxel‐wise analysis revealed a spatial pattern of higher R2 for higher iron levels, particularly in gray matter (Fig. 1). The pattern included basal ganglia structures such as the globus pallidus and putamen, and cortical regions including the precentral, postcentral and cuneus cortex (Fig. 1). Higher lobar and total WMH burden were also associated with higher iron levels (Fig. 2). No negative associations were observed.

**Conclusion:**

This investigation combined ex‐vivo MRI, neuropathology and mass spectrometry in a community‐based older adults and showed that higher iron levels were independently associated with higher R2 in gray matter and higher WMH burden.